# The Arabidopsis RNA Polymerase II Carboxyl Terminal Domain (CTD) Phosphatase-Like1 (CPL1) is a biotic stress susceptibility gene

**DOI:** 10.1038/s41598-018-31837-0

**Published:** 2018-09-07

**Authors:** Louise F. Thatcher, Rhonda Foley, Hayley J. Casarotto, Ling-Ling Gao, Lars G. Kamphuis, Su Melser, Karam B. Singh

**Affiliations:** 1 CSIRO Agriculture and Food, Centre for Environment and Life Sciences, Floreat, Western Australia Australia; 2 Centre for Crop and Disease Management, Department of Environment and Agriculture, Curtin University, Bentley, Western Australia 6102 Australia; 3 Present Address: Su Melser, INSERM U1215, Université de Bordeaux, NeuroCentre Magendie, Bordeaux, France

## Abstract

Crop breeding for improved disease resistance may be achieved through the manipulation of host susceptibility genes. Previously we identified multiple Arabidopsis mutants known as *enhanced stress response1* (*esr1*) that have defects in a KH-domain RNA-binding protein and conferred increased resistance to the root fungal pathogen *Fusarium oxysporum*. Here, screening the same mutagenized population we discovered two further *enhanced stress response* mutants that also conferred enhanced resistance to *F*. *oxysporum*. These mutants also have enhanced resistance to a leaf fungal pathogen (*Alternaria brassicicola*) and an aphid pest (*Myzus persicae*), but not to the bacterial leaf pathogen *Pseudomonas syringae*. The causal alleles in these mutants were found to have defects in the ESR1 interacting protein partner RNA Polymerase II Carboxyl Terminal Domain (CTD) Phosphatase-Like1 (CPL1) and subsequently given the allele symbols *cpl1-7* and *cpl1-8*. These results define a new role for CPL1 as a pathogen and pest susceptibility gene. Global transcriptome analysis and oxidative stress assays showed these *cpl1* mutants have increased tolerance to oxidative stress. In particular, components of biotic stress responsive pathways were enriched in *cpl1* over wild-type up-regulated gene expression datasets including genes related to defence, heat shock proteins and oxidative stress/redox state processes.

## Introduction

The term susceptibility gene was first coined several decades ago to describe the phenomenon of plant genes required for susceptibility to specific pathogens (reviewed in Eckardt^1^). Modification or removal of these genes led to enhanced resistance (reviewed in Van Schie and Takken^2^). Van Schie and Takken proposed three distinct classes of susceptibility genes based on their involvement in different stages of pathogen infection. In order of the infection process these are: (1) compatibility and pathogen establishment, (2) modulation of host defences, and lastly (3) mechanisms facilitating pathogen proliferation and sustenance.

The second class of susceptibility genes contains many that encode negative regulators that act to keep the plant defence response under control and ready for release upon pathogen detection. The overwhelming majority of these genes have been assessed for susceptibility to leaf diseases. In most cases, the enhanced or constitutively activated defences in this class of mutants leads to deleterious pleiotropic effects^2^. For example, in Arabidopsis the cellulose synthase (CESA3) mutant *constitutive expression of VSP1* (*cev1*), the MAP kinase mutant *mpk4*, or the *constitutive expression of PR genes 5* mutant *cpr5*, exhibit increased resistance to leaf powdery or downy mildew pathogens but suffer from spontaneous lesions, early senescence, dwarfing, and/or increased susceptibility to the necrotrophic leaf fungus *Alternaria brassicicola*^3–9^. The discovery of root pathogen susceptibility genes is less reported, inherently due to the more difficult nature of studying root diseases. However, the intractable nature of most root diseases (e.g. long-term persistence in soil/stubble and/or lack of dominant *Resistance* genes) compels a strong case for the discovery of root pathogen susceptibility genes. These can however also confer deleterious side-effects. For example, the Mediator complex subunit mutant *med25/pft1*, the JA-coreceptor mutant *coronatine insenstive1* (*coi1*), the MAP kinase phosphatase mutant *mkp2*, and the auxin transporter mutant *wat1*, exhibit increased resistance to root fungal or bacterial wilts (e.g. *Fusarium oxysporum*, *Verticillium dahliae*, *Ralstonia solanacearum*) but suffer from increased susceptibility to leaf necrotrophs (e.g. *A*. *brassicicola*, *Botrytis cinerea*), early senescence, delayed flowering or reduced seed set^10–17^.

We previously discovered a root pathogen susceptibility gene that lacked any observable deleterious pleiotropic effects. The gene encoded a K homology (KH) domain RNA-binding protein and several mutant alleles, termed *enhanced stress response1* (*esr1*), conferred increased resistance to the root fungal pathogen *F*. *oxysporum*, but lacked any observable deleterious defects in growth or development^18^. Interestingly, the *esr1* mutants also conferred increased tolerance to abiotic stress with other alleles identified from abiotic stress screens (*regulator of C Repeat Binding Factor* (*CBF*) *gene expression 1*, *rcf3-1*; *shiny1*, *shi1*; *high osmotic stress gene expression 5*, *hos5-1*)^19–21^. While *ESR1* encodes a negative regulator of *F*. *oxysporum* resistance, the *esr1* mutants do not have constitutive or enhanced expression of defensive components that largely categorize class two susceptibility genes. On the contrary, these mutants are suppressed in components of jasmonate (JA) hormone-mediated responses. This included both defensive and metabolic responses, and was not associated with up-regulated salicylic acid (SA) defences typical of antagonistic JA-SA crosstalk^22^. This suggests ESR1 spans both class two and three susceptibility gene categories.

The *esr1* mutants were discovered from an ethyl methansulfonate (EMS) mutagenized population of plants containing a root-specific stress marker (*GLUTATHIONE S-TRANSFERASE PHI8* (*GSTF8*) *promoter: LUCIFERASE reporter*)^18,23^. Non-destructive *in vivo* imaging of *GSTF8:LUC* activity in Arabidopsis roots has been used to successfully follow root-specific stress responses to biotic signals of both endogenous (e.g. SA, reactive oxygen species) and exogenous origin (e.g. the root fungal pathogen *Rhizoctonia solani*)^24–26^. Other mutants identified from the *GSTF8:LUC* screen included *disrupted in stress responses1* (*dsr1*), encoding a positive regulator of plant defences^23^. This mutant exhibited a loss of SA inducible *GSTF8:LUC* activity and increased susceptibility to several fungal and bacterial pathogens. The causal mutation was encoded within a subunit of the mitochondrial energy machinery (complex II subunit SDH1-1) and resulted in a reduction in induced reactive oxygen species production (ROS) from mitochondria^23^.

To identify other susceptibility genes, we extend on our *esr* mutant collection to identify another class two susceptibility gene, an ESR1/KH domain RNA-binding interacting protein partner termed Enhanced Stress Response3/RNA Polymerase II C-Terminal Domain (CTD) Phosphatase-Like1 (CPL1). The Arabidopsis genome encodes four *CTD phosphatase-like* (*CPL*) genes, with *CPL1* and *CPL2* being plant specific^27,28^. Arabidopsis CPL1 has been demonstrated to function in multiple RNA processing roles including RNA Pol II CTD dephosphorylation, mRNA capping, pre-mRNA splicing and RNA decay, and physically interacts with several transcription factors or co-regulators such as HOS5/RCF3/ESR1 and serine-arginine rich splicing factors^20,21,29–33^.

The *cpl1* mutants we isolated displayed strong resistance to *F*. *oxysporum*, as well as increased resistance to a leaf fungal pathogen and insect pest, but not to a bacterial leaf pathogen. Whole transcriptome sequencing of the strongest disease suppressive *cpl1* allele identified up-regulated expression of genes involved in responses to biotic and abiotic stress including control of oxidative stress. The *cpl1* alleles and an independent *cpl1-1* T-DNA insertion mutant were functionally tested for altered oxidative stress responses and found to exhibit reduced sensitivity to the chemical oxidative stress inducer methyl viologen. Combined, these results define CPL1 as a pathogen and pest susceptibility gene where it acts as a negative mediator on components of defence and redox state processes.

## Results

### Identification of *esr3* mutants

**Figure 1:**
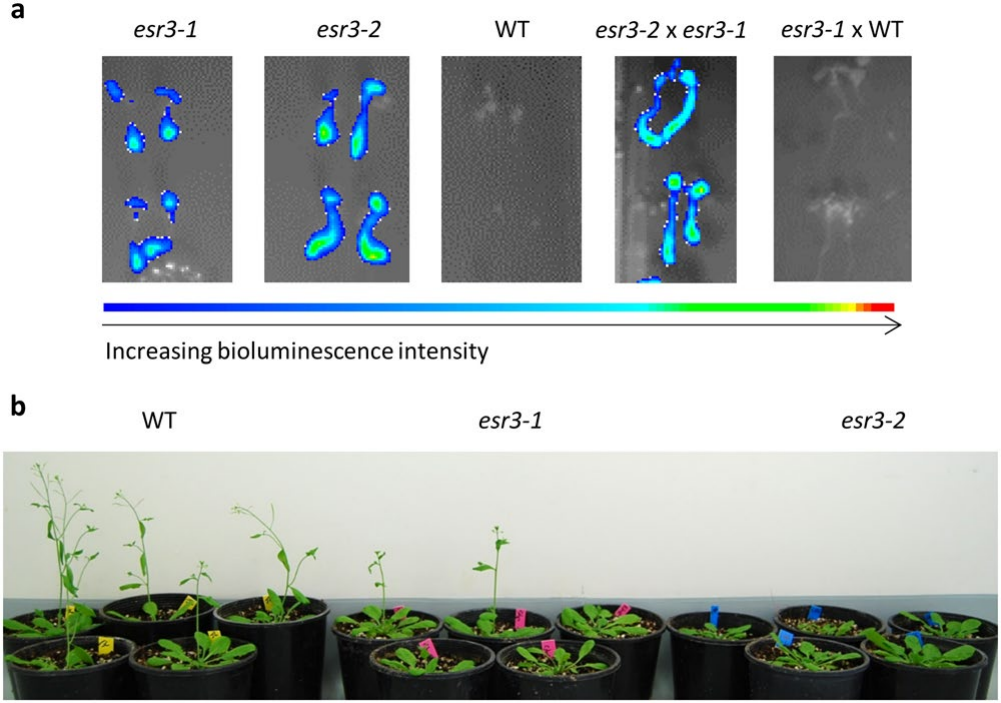
Identification of two *esr3* alleles. (**a**) *esr3-1* and *esr3-2* mutants were crossed and F_1_ progeny screened for complementation of the constitutive *GSTF8:LUC* phenotype. A cross to wild-type (WT) *GSTF8:LUC* is included as a negative control. Intensity of bioluminescence ranges from blue to red as depicted in the intensity ruler. (**b**) *esr3* mutants are delayed in flowering with a stronger phenotype observed for *esr3-2*. Shown are representative plants at 28 days of age grown under long day conditions.

In the previous screen that identified *esr1* mutants^18^, over 50 other constitutively expressing *GSTF8:LUC* mutants were identified including one labelled *esr3-1* with some of the highest levels of basal root-expressed promoter expression. Allelism tests revealed this mutant was not allelic to *esr1-1*. The *esr3-1* mutant was observed to exhibit a delayed flowering phenotype which was replicated in a stronger form by another *esr* mutant with a stronger *GSTF8:LUC* phenotype. F_1_ allelism tests revealed that the second, stronger mutant was an allele of *esr3-1* and subsequently labelled *esr3-2* (Fig. 1). Apart from the delayed flowering phenotype, the *esr3* mutants were otherwise phenotypically normal. No difference in vegetative biomass pre wild-type flowering was recorded (Welch’s test). For cloning and heritability studies, both mutants were out-crossed to the Landsberg *erecta* ecotype (Ler). All F_1_ plants showed the wild-type phenotype, and all F_2_ plants displayed a ~3:1 (wild-type:mutant) segregation (*esr3-1* 57:23, χ^2^ test *p* = 0.44; *esr3-2* 200:87, χ^2^ test p = 0.12). These results suggest both *esr3-1* and *esr3-2* are recessive mutations in a single nuclear gene.

### *ESR3* Encodes a RNA polymerase II (Pol II) carboxyl terminal domain (CTD) phosphatase-like 1 (CPL1) protein

**Figure 2:**
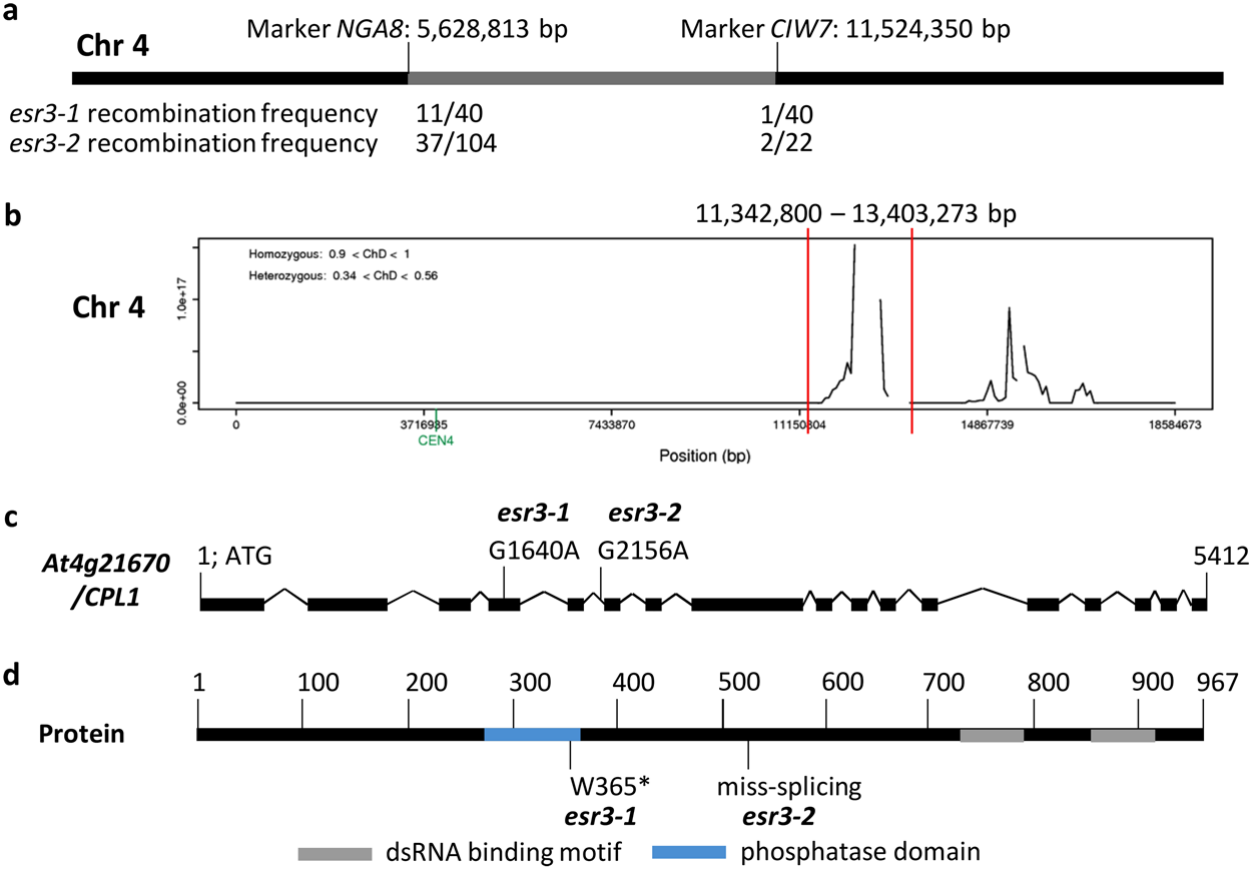
Molecular cloning of *esr3* alleles. (**a**) Fine mapping of *esr3-1* and *esr3-2* narrowed their mutations to chromosome 4. Shown are recombination events over total number of chromosomes analysed for each flanking marker. (**b**) Next Generation Mapping was applied to the *esr3-1* mapping population by sequencing homozygous *esr3-1* F_2_ plants, and processing SNPs that deviated from the Arabidopsis TAIR10 genome reference sequence through the NGM tool http://bar.utoronto.ca/ngm/^59^. The tool narrowed the *esr3-1* locus on chromosome 4 as indicated by peaks on the y-axis (ratio of homozygous to heterozygous signals). The first peak (bordered by two red bars) contained six candidate mutations, two (At4g21460; At4g21670) of which localised to the fine mapped region. The second peak resided outside the fine-mapped region. (**c**) Wild-type (WT), *esr3-1* and *esr3-2* genomes were sequenced and inspected for SNP differences within the mapped loci, identifying one candidate, *At4g21670* (*CPL1*). Structure of the *At4g21670/CPL1* gene with *esr3* mutations indicated. Filled boxes indicate exons, joining lines indicate introns. Positions are relative to the start codon. (**d**) Domain structure of the At4g21670/CPL1 protein and position and predicted nature of the *esr3* mutations indicated. Positions are relative to the first methionine.

We undertook two complementary map based cloning approaches to identify the causal *esr3* mutations. Firstly, genetic mapping using *esr3-1* and *esr3-2* F_2_ mapping populations localised the causal mutations to a 5.9 Mbp region of chromosome 4 (Fig. 2a). Secondly, Next Generation Mapping on *esr3-1* also narrowed the *esr3-1* locus to chromosome 4 and identified six candidate mutations in genes *At4g21460*, *At4g21670*, *At4g21690*, *At4g22890*, *At4g24170* and *At4g24730* (Fig. 2b, Supplemental Figure S1). Two of these mutations (*At4g21460* and *At4g21670*) localised to the *esr3-1* and *esr3-2* fine mapped region and interestingly included the ESR1/HOS5/RCF3 interacting protein CPL1 (*At4g21670/CPL1*). We sequenced the *CPL1* gene *At4g21670* from *esr3* mutant lines that had been backcrossed to wild-type plants three times to remove any additional unwanted EMS-induced mutations. Single nucleotide changes were identified in the *CPL1* gene from both *esr3* mutants but not from wild-type plants. The SNPs identified were a G1640A nucleotide change in *esr3-1* resulting in a stop codon change (W365*) within the CPL1 phosphatase domain, and a G2156A nucleotide change in *esr3-2* within the splicing acceptor site of the fifth intron which would be predicted to result in aberrant transcripts lacking the dsRNA binding motif sequences. To determine if the *cpl1* mutations were solely responsible for the observed *esr3* phenotypes and no other residual EMS-induced mutations, whole genome sequencing of wild-type (*GSTF8:LUC*), *esr3-1* and *esr3-2* individuals was conducted. Inspection for SNP differences within the 5.9 Mbp mapped loci only identified one candidate gene, *At4g21670/CPL1*, that contained SNPs residing in both *esr3-1* and *esr3-2*. The position of these SNPs, supported 100% by over 30 reads, and their predicted effect on CPL1 protein structure is highlighted in Fig. 2c,d, where one results in a premature stop and the other in a mis-splicing site from the EMS mutations.

It was recently reported several independent *LUCIFERASE* reporter hyperexpression based forward genetic screens commonly identified mutations in both *ESR1*/*HOS5*/*RCF3* and *CPL1* genes^34^. CPL1 encodes RNA Polymerase II carboxyl terminal domain (CTD) phosphatase-like 1 (CPL1) and interacts with and is required for ESR1/HOS5/RCF3 recruitment to the nucleus^20,21,31^. The protein contains a phosphatase domain and two double-stranded RNA binding motifs, and functions in transcriptional and post-transcriptional metabolism of mRNA, including RNA capping efficiency and decay of abnormally spliced transcripts, as well as dephosphorylation of the CTD of RNA Pol II^20,21,29–33^. Koiwa and Fukudome^34^ suggest *LUCIFERASE mRNA* is also a target of CPL1-dependent RNA decay and explains why mutations in CPL1 and its binding partner ESR1/HOS5/RCF3 are responsible for *LUCIFERASE* reporter hyperexpression phenotypes. The *esr3* mutants and published *cpl1* mutants^35,36^ share common LUCIFERASE reporter hyperexpression and delayed flowering phenotypes.

**Figure 3:**
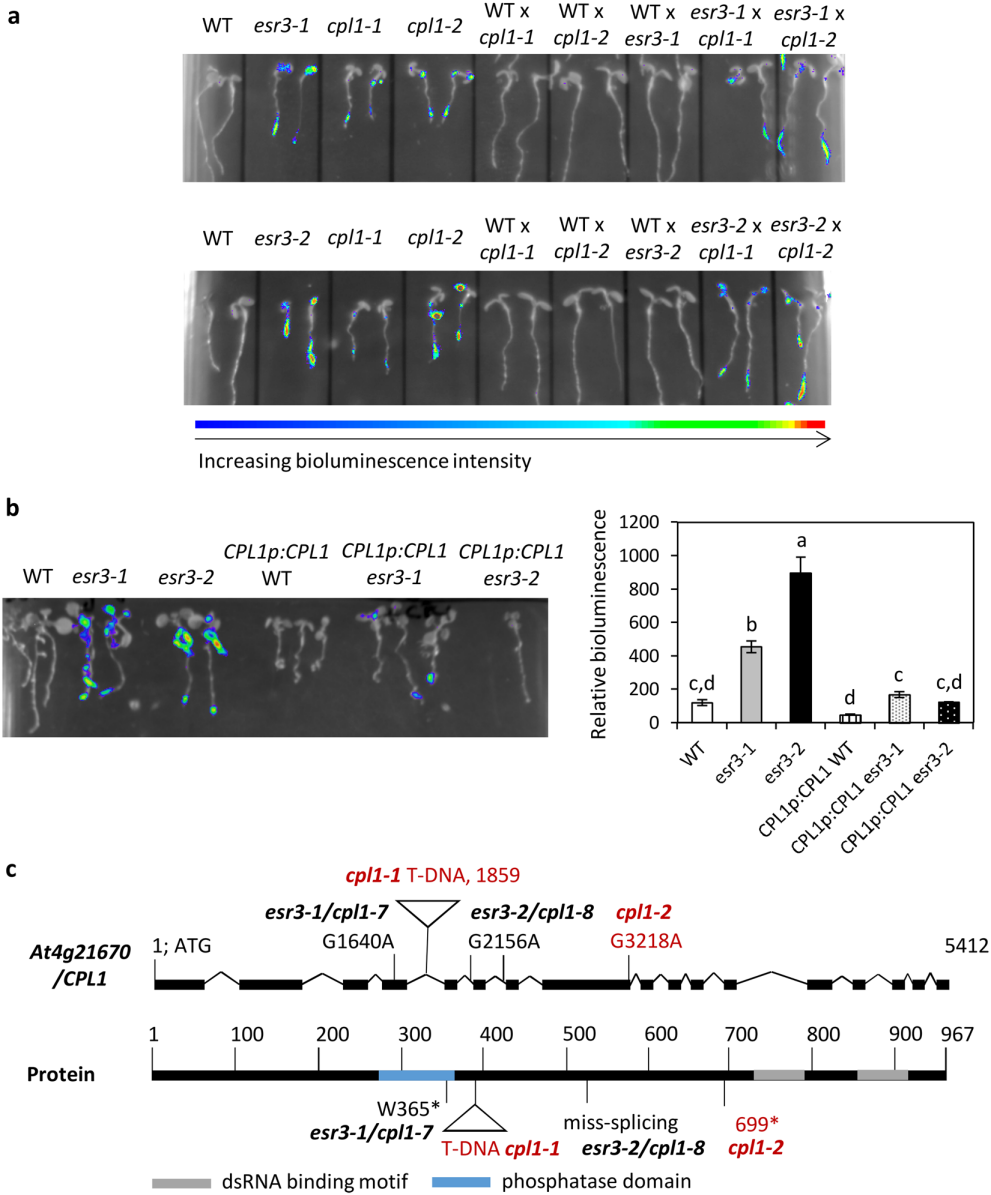
Genetic and molecular complementation of *esr3* phenotypes with *CPL1*. (**a**) Genetic complementation between *esr3* mutants and a *CPL1* At4g21670 T-DNA insertion line (*cpl1-1*) or an EMS mutant line (*cpl1-2*, also known as *fry2-1*). The homozygous recessive mutants were crossed and F_1_ progeny screened for complementation of the *GSTF8:LUC* phenotypes. Intensity of bioluminescence ranges from blue to red as depicted in the intensity ruler. (**b**) Molecular complementation of the *esr3-1* and *esr3-2* mutations by the wild-type At4g21670 *CPL1* gene driven by its native promoter (*CPL1p:CPL1*). Values are average luminescence counts per seedling ± SE (n = 6, except for *CPL1p:CPL1* in *esr3-2* where only one transformant was obtained) *P* < 0.05, all pairs Student’s *t*-test. (**c**) CPL1 gene and protein structure highlighting the *cpl1* mutations. Details are as in Fig. 2.

Genetic complementation assays were conducted by crossing *esr3* and wild-type *GSTF8:LUC* plants with published *cpl1-1* or *cpl1-2* mutants^35,36^ and assessing F1 phenotypes. The *cpl1-1* and *cpl1-2* (*fiery2-1*/*fry2-1*) mutants are homozygous recessive T-DNA insertional or EMS mutants respectively that result in hyperexpression of the osmotically regulated promoter construct *RD29A:LUC* in response to cold, salt (NaCl) or abscisic acid (ABA) treatments, and are delayed in flowering (Supplemental Figure S2a–c). Both *esr3* mutants display a *cpl1-2* increased salt stress tolerance phenotype (Supplemental Figure S2d–e). Unlike F_1_s from wild-type *CPL1* crossed with *esr3* or *cpl1* which displayed no constitutive luciferase expression (*WT:esr3-1*; *WT:esr3-2*; *WT:cpl1-1*; *WT:cpl1-2*), the F_1_ *esr3* and *cpl1* crossed plants retained their *esr3* and *cpl1* phenotypes and exhibited constitutive bioluminescence (Fig. 3a), supporting the mapping and sequencing results that mutations in *At4g21670/CPL1* are responsible for the *esr3* mutant phenotypes.

We also conducted molecular complementation assays by introducing the *CPL1*/*At4g21670* gene under the control of its endogenous promoter (~1 kb) into the *esr3* backgrounds. The introduction of this construct (*CPL1p:CPL1*) reduced the *esr3-1* constitutive *GSTF8:LUC* expression and restored the wild-type phenotype (Fig. 3b). The *CPL1* construct also reduced constitutive *GSTF8:LUC* expression in *esr3-2* however, only one transformant was obtained due to low pollen production in this mutant. Combined, these results point solely towards mutations in *CPL1* responsible for the *esr3-1* and *esr3-2* phenotypes. In light of other *cpl1* alleles, herein and going forward we propose the allele symbols *cpl1-7* and *cpl1-8* be assigned to *esr3-1* and *esr3-2* respectively. In summary, a schematic representation of the *cpl1* alleles assessed in this study are shown in Fig. 3c.

### The *cpl1* mutants confer increased resistance to specific fungal pathogens and insect pests

**Figure 4:**
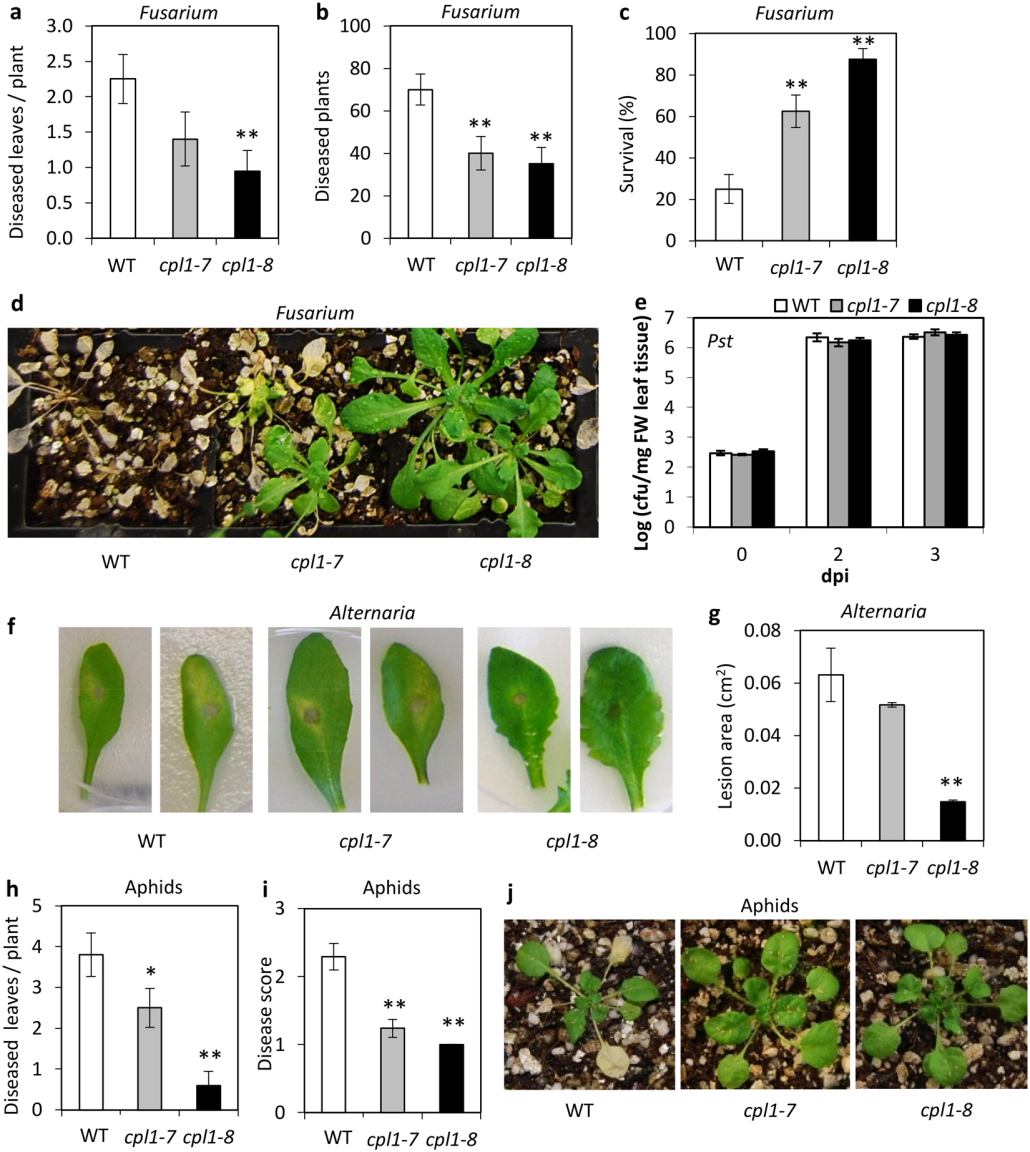
*cpl1* mutants have increased resistance/tolerance to fungal pathogens and an insect pest. (**a**–**d**) Disease phenotypes of *F*. *oxysporum* inoculated plants with (**a**) diseased leaves and (**b**) diseased plants at 7 days post inoculation (dpi) with the pathogen, (**c**–**d**) survival, and representative images of plants at 21 dpi. Values are averages ± SE (n = 40). (**e**) *Pseudomonas syringae* (*Pst*) DC3000 growth on inoculated leaves. Values are averages ± SE of 3 biological replicates consisting of pools of 4 leaves. (**f**–**g**) *A*. *brassicicola* induced lesions at 3 dpi with (**f**) representative images of leaves and (**g**) size of lesions. Values are averages ± SE of 3 biological replicates consisting of lesions measured from 5 inoculated leaves per plant. (**h**–**j**) Disease phenotypes of *M*. *persicae* infested plants with (**h**) diseased leaves (**i**) disease score of symptomatic leaves and (**j**) representative images of plants 14 dpi. Disease scores ranged from 1 (minimum symptoms) to 4 (whole leaf necrotic). Values are averages ± SE (n = 10). Asterisks indicate values that are significantly different (**P < 0.01, *P < 0.05 Student’s *t-*test) from wild-type (WT). Similar results were obtained in independent experiments.

The *esr1-1* mutant exhibits increased resistance to the root-infecting pathogen *Fusarium oxysporum*^18^, while the *dsr1* mutant exhibits increased susceptibility to the root-infecting pathogen *Rhizoctonia solani*^23^. We therefore tested if our *cpl1* mutants conferred increased resistance to these root pathogens. Both a reduction in disease symptom development (1.8 and 2-fold less respectively than wild-type) and increased survival (2.5 and 3.5-fold respectively over wild-type) was observed for both *cpl1-7 and cpl1-8* compared to wild-type when inoculated with *F*. *oxysporum* (Fig. 4a–d). Interestingly, the *cpl1-8* mutant which possessed enhanced *GSTF8:LUC* expression and a stronger delay in flowering relative to *cpl1-7*, also exhibited stronger disease resistance. We validated the increased *F*. *oxysporum* disease resistance phenotypes in the independent *cpl1-1* and *cpl1-2* mutants (Supplemental Figure S3). No significant difference in disease development between mutants and wild-type were observed when inoculated with *R*. *solani* (Supplemental Table S1). These results identify *CPL1* as a *F*. *oxysporum* susceptibility gene and suggest the resistance response observed in *cpl1* mutants may be linked to the plants’ specific defence response against *F*. *oxysporum* rather than towards the general nature of root-infecting fungal pathogens.

We were interested to determine if *cpl1* mediated resistance against a root pathogen extended to resistance against leaf pathogens. Wild-type, *cpl1-7* and *cpl1-8* plants were inoculated with the leaf bacterial pathogen *Pseudomonas syringae* cv. *tomato* (*Pst*) DC3000) or the leaf fungal pathogen *Alternaria brassicicola*. No significant difference in *Pst* disease progression was observed, but smaller *A*. *brassicicola* induced lesions and leaf chlorosis/senescence were observed on *cpl1-8* leaves and to a lesser but not significant level on *cpl1-7* (Fig. 4e–g). The green peach aphid *M*. *persicae* is a major pest of many crops and is known to induce senescence responses in Arabidopsis (reviewed in de Vos *et al*.^37^). Upon presentation of green peach aphids we found *cpl1* mutants had less aphid-induced chlorotic/necrotic leaves compared to wild-type (Fig. 4h–j). No significant difference in total aphid weights from *cpl1* mutant or wild-type infested plants was recorded, suggesting the *cpl1* mutations did not change the aphid population but reduced host aphid-induced symptom development. Overall, the pest and disease phenotypes suggest chlorosis and senescence responses might be reduced in *cpl1* mutants, and that other aspects of plant defence are potentially also altered.

### *cpl1-8* whole genome transcript analysis reveals up-regulation of genes involved in plant defence, heat shock and redox processes

To identify genes that might be controlling the *cpl1* phenotypes we conducted a RNA sequencing (RNA-seq) experiment between wild-type and the stronger *cpl1-8* allele. To capture inherent transcript changes resulting from *CPL1* disruption, we chose to analyse basal differences rather than to follow changes specific to a particular pathogen or pest stress treatment. Between 56 and 66 million paired-ends reads (100 bp) were generated for three biological replicates derived from both genotypes and mapped with an alignment rate of 92–94.5% to the TAIR10 genome reference with estimated exome coverage of ~100 times. Overall, 717 genes were significantly differentially regulated ≥2-fold in *cpl1-8* compared to wild-type (Benjamini-Hochberg correction for multiple-testing based on a False Discovery Rate (FDR) < 0.05) (Supplemental Tables S2 and S3). To gain insight into the functions of these genes we performed Gene Ontology (GO) term enrichment analysis. Within the up-regulated dataset (401 differentially expressed genes (DEGs)), 44 biological process GO categories were significantly overrepresented and enriched in processes associated with response to stimulus (chemical, abiotic, hormone, biotic), oxidative stress and defence (Supplemental Figure S4).

**Figure 5:**
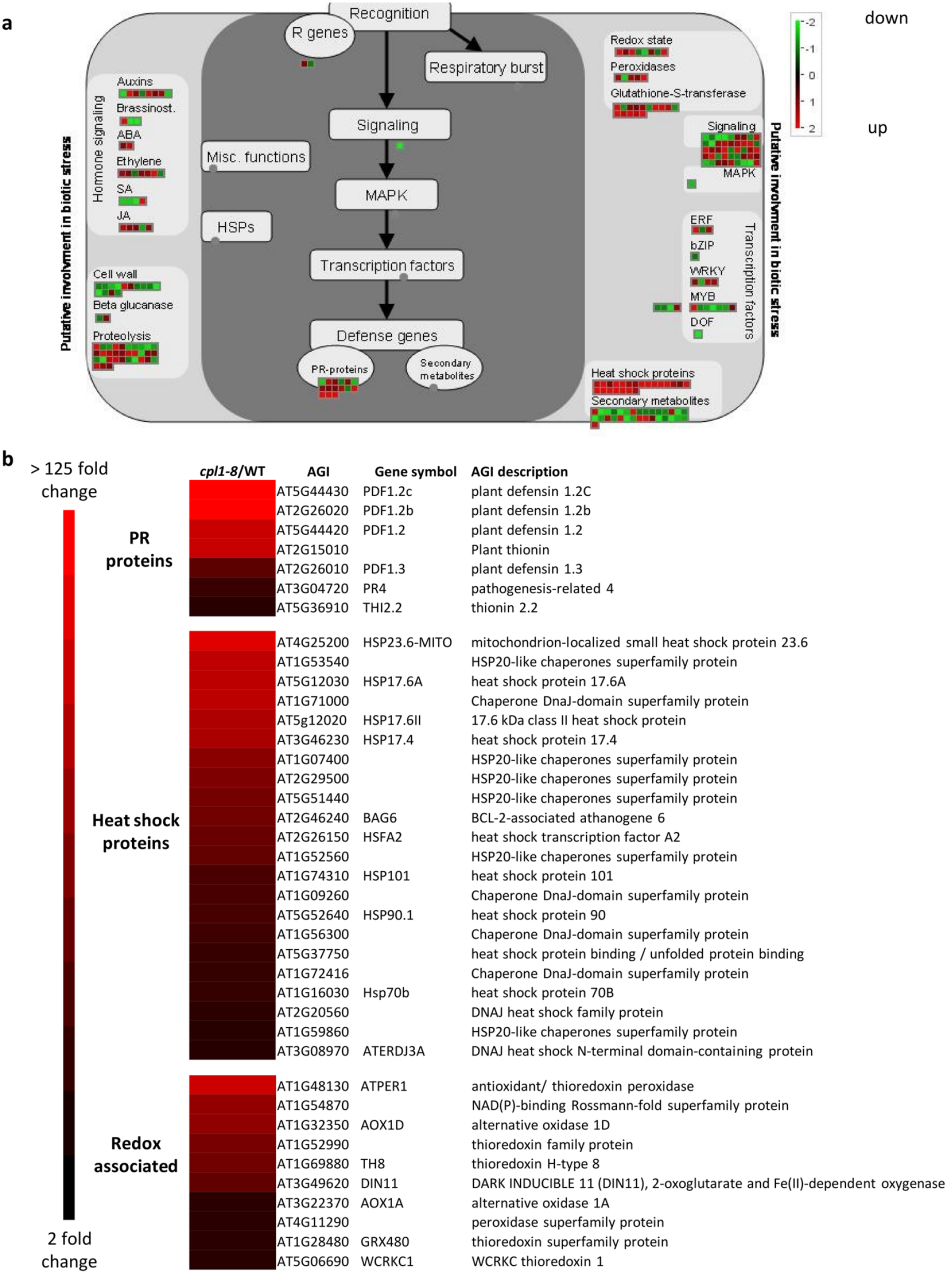
Defence-associated genes are enriched in *cpl1-8* up-regulated genes. (**a**) Log2 fold changes in *cpl1-8*/WT gene expression associated with biotic stress as determined by MapMan bin terms with up-regulated and down-regulated genes represented by red or green squares respectively. (**b**) RNAseq expression heat maps of PR, heat shock and redox associated protein categories enriched in *cpl1-8* up-regulated genes. FC: fold change *cpl1-8*/WT.

MapMan pathway analysis^38^ supported the GO term analysis with defence-associated hormone signalling, pathogenesis related (*PR*), heat shock, and oxidative stress and redox control processes up-regulated in *cpl1-8* (Fig. 5a). This included for example the JA-regulated *PR* marker genes *PDF1*.*2*, *PR4*, and *PR13* (*THIONIN*), JA biosynthesis genes (*AOC2*), heat shock factors (*HSFA2*) and heat shock proteins (*HSP17*, *HSP70*, *HSP90*), and redox responsive *GSTs* and *THIOREDOXINS* such as *GSTF6* and *GSTF7* (Fig. 5b). We validated the expression of representative members of these genes by qRT-PCR (Supplemental Figure S5). Combined, these results suggest CPL1 has a role in the negative regulation of defence and redox responses.

### *CPL1* and *ESR1* are involved in the expression of a subset of genes responsive to biotic stress

**Figure 6:**
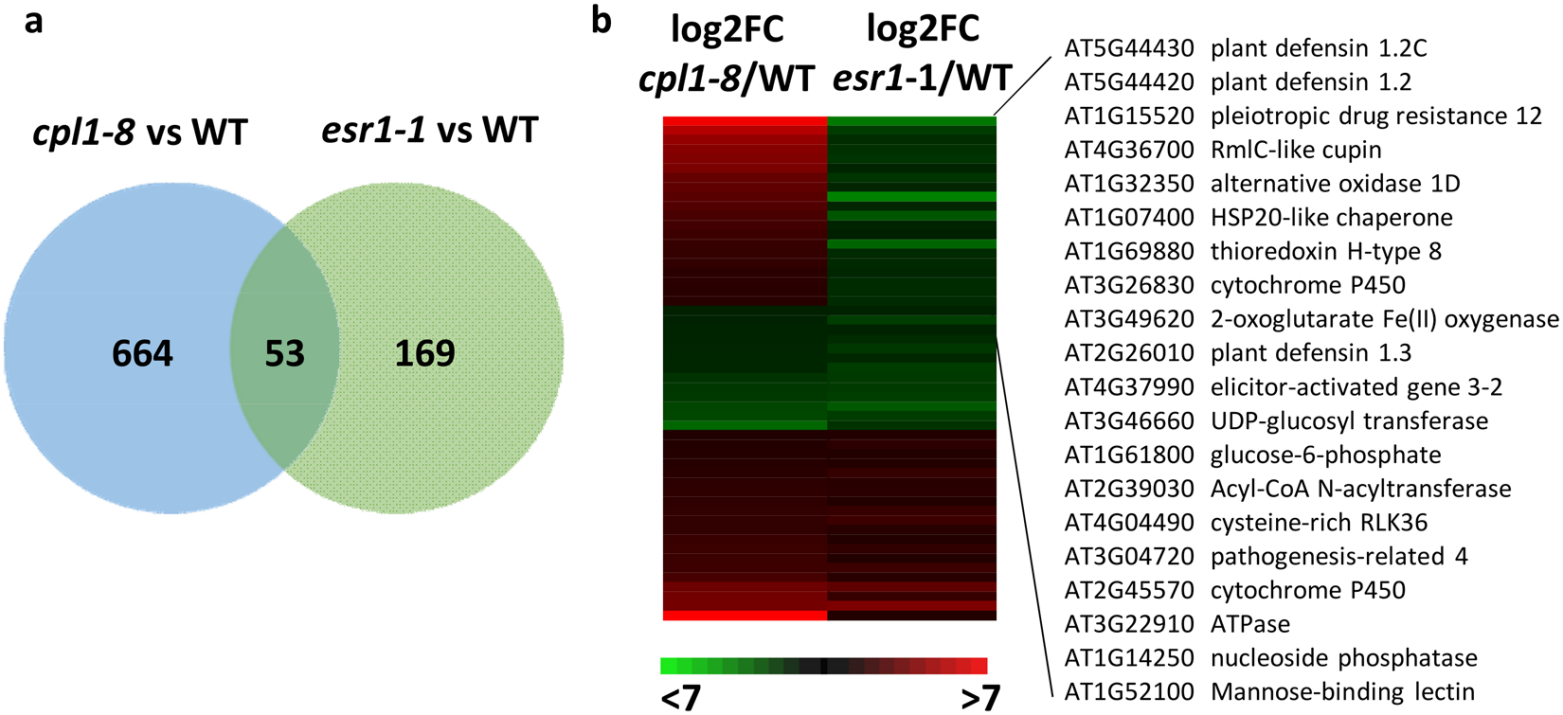
*CPL1* and *ESR1/HOS5/RCF3* regulate a subset of stress-responsive genes. (**a**) Venn diagram of the genes differentially expressed ≥2-fold between *cpl1-8* and WT or *esr1-1* and WT. (**b**) Heat map of the 53 overlapping genes. The heat map shows up-regulation (red) or down-regulation (green) relative to wild-type (WT).

As CPL1 and the KH-domain RNA binding ESR1/HOS5/RCF3 proteins interact^20,21^, we hypothesized they may co-regulate similar sets of genes. To test this we compared *cpl1-8* vs wild-type and *esr1-1* vs wild-type^18^ differentially expressed genes (DEGs). Firstly, 3-fold more DEGs were identified in the *cpl1-8* dataset (717) compared to *esr1-1* (222). A comparison of these datasets amongst each other found approximately 24% of *esr1-1* DEGs and 7% of *cpl1-8* DEGs overlapped (Fig. 6a) and were enriched in GO categories relating to multi-organism processes and response to biotic stimulus. Interestingly, 38% of these overlapping genes showed opposite expression profiles being up-regulated in *cpl1-8* but down-regulated in *esr1-1* (Fig. 6b). These were enriched in response to stimulus (biotic, chemical), stress or defence response GO biological process categories and included *PDF1*.*2*, *PDF1*.*3*, *PR4* and *ELICITOR-ACTIVATED GENE 3-2*. This is consistent with our findings that CPL1 has a role in negative regulation of defences while ESR1 is a positive regulator. Overall, these results suggest CPL1 and ESR1/HOS5/RCF3 play roles in the expression of a subset of stress-responsive genes.

### *cpl1* mutations confer increased sensitivity to JA

**Figure 7:**
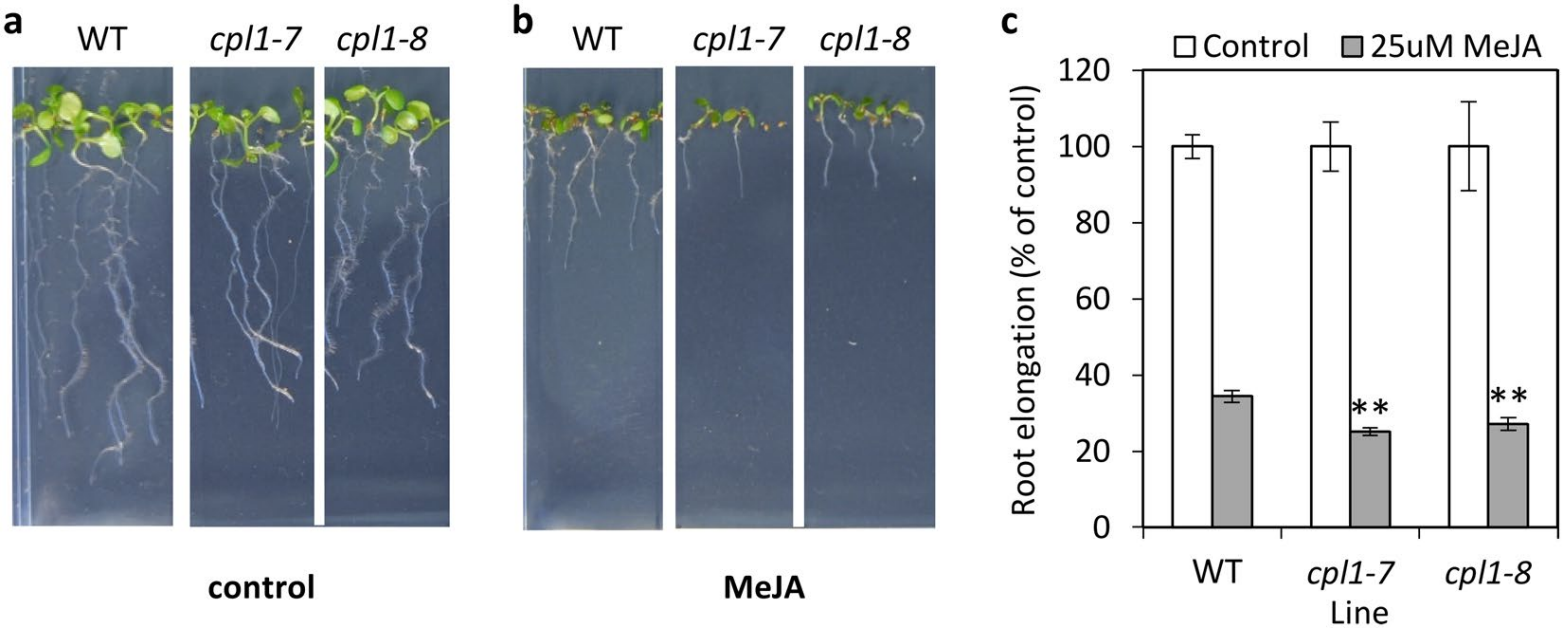
*cpl1* alleles have increased JA sensitivity. (**a**–**c**) Sensitivity of wild-type (WT) and *cpl1* seedlings to JA was determined by MeJA inhibition of root growth on (**a**) control media or (**b**) media containing MeJA (25 uM). (**c**) Root elongation of each line when grown on MeJA was calculated as a percentage relative to their root length on the control. Shown is the average ± SE of 5 biological replicates consisting of pools of 10 seedlings. Asterisks indicate values that are significantly different (**P < 0.01, Student’s *t*-test) from WT. Similar results were obtained in an independent experiment.

The up-regulation of JA-regulated defensive genes in our RNA-seq data (e.g. *PDF*1.2, *PR4*) prompted us to assess JA sensitivity in the *cpl1* mutants via MeJA root inhibition assays. The roots of *cpl1* seedlings showed a small, but significant, increase in sensitivity to MeJA treatment compared to wild-type roots (Fig. 7), and this was more pronounced in seedlings treated with a higher concentration of MeJA (Supplemental Figure S6).

### *cpl1* mutations confer reduced sensitivity to oxidative stress

**Figure 8:**
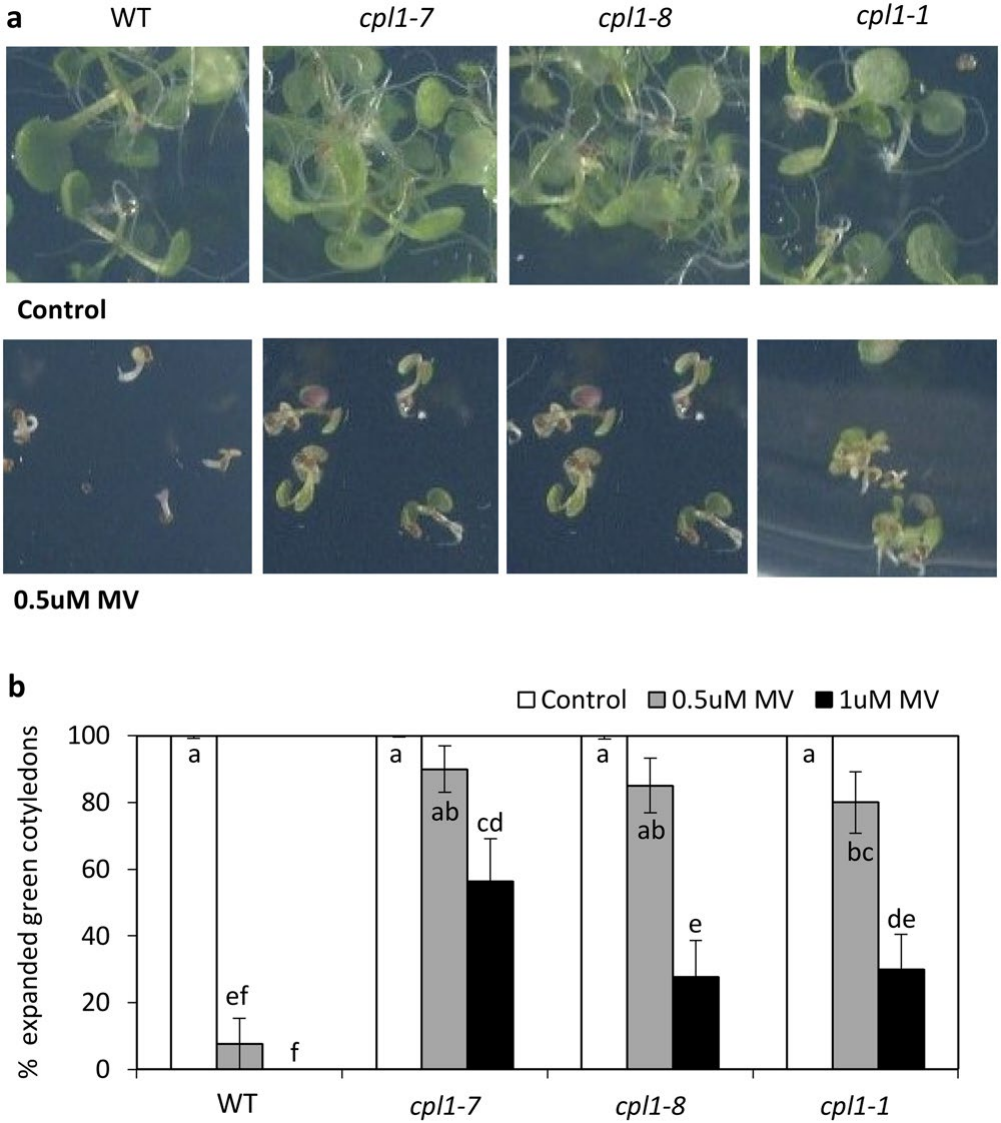
*cpl1* alleles have reduced sensitivity to oxidative stress inducer methyl viologen. Sensitivity of wild-type (WT), *cpl1-1*, *cpl1-7* and *cpl1-8* alleles to methyl viologen (MV) was determined by germination on control media or media containing 0.5 uM or 1 uM MV MeJA. (**a**) Images of representative seedlings grown on MV and (**b**) the percentage of green, fully emerged cotyledons determined at 10 days post treatment. Shown is the average ± SE (n = 13–20). *P* < 0.05, all pairs Student’s *t*-test. Similar results were obtained in an independent experiment.

We noted genes related to redox control and oxidative stress responses were enriched in *cpl1-8* DEGs. To determine whether CPL1 has a role in regulating oxidative stress responses, we treated wild-type and *cpl1* alleles with the superoxide generator methyl viologen (Paraquat). Following 0.5 µM methyl viologen treatment, 80–90% of *cpl1* seedlings had developed fully expanded cotyledons compared to only 8% of wild-type seedlings (Fig. 8). These results are consistent with our observation that CPL1 negatively regulates oxidative stress tolerance.

## Discussion

We set out to identify novel regulators of pathogen resistance and from an EMS mutagenized Arabidopsis population containing the *GSTF8:LUC* root-stress marker we identified *cpl1-7* and *cpl1-8* as two additional *cpl1* alleles and define RNA Polymerase II C-Terminal Domain (CTD) Phosphatase-Like1 (CPL1) as a new pathogen and pest susceptibility gene. We determined CPL1 belongs to a class of susceptibility genes encoding negative regulators, and potentially confers a broad role in susceptibility to different classes of pathogens and insect pests as we demonstrated against both root and leaf fungal pathogens and with aphid pests.

Sequencing of the *CPL1* gene *At4g21670* from the *cpl1* mutants identified a G1640A nucleotide change in *cpl1-7* resulting in a stop codon change within the CPL1 phosphatase domain, and a G2156A nucleotide change in *cpl1-8* within the splicing acceptor site of the fifth intron (Fig. 2c,d). The later mutation would be predicted to result in aberrant transcripts lacking the dsRNA binding motifs. Assessment of RNA-seq data confirmed the fifth *cpl1-8 CPL1* intron is not correctly spliced out (Supplemental Figure S7) and is predicted to encode a premature stop codon, generating a predicted truncated protein that is likely non-functional. The *cpl1-1* and *cpl1-2/fry2-1* alleles were both identified through abiotic (cold, drought, salt, ABA) stress-responsive *RESPONSIVE TO DESICCATION 29A* (*RD29A*) promoter screens and exhibit increased tolerance to salt stress, iron deficiency, cadmium toxicity, and to abscisic acid (ABA) during seed germination^35,36,39^. Other *cpl1* alleles have been identified through similar luciferase reporter screens using salt (*SULFOTRANSFERASE SOT12; shi4*) or wound responsive JA-biosynthesis promoters (*FATTY ACID DESATURASE 7*, *FAD7; cpl1-3*)^20,35,36,40^. Matsuda *et al*.^40^ identified *CPL1* as a negative regulator of JA-biosynthesis promoter activity (*FAD7*; *OXOPHYTODIENOATE-REDUCTASE* 3, *OPR3*; *ALLENE OXIDE SYNTHASE*, *AOS*).

The CTD of the largest RNA Pol II subunit plays a critical role in eukaryotic transcriptional control where specific, reversible CTD modifications coordinate the recruitment of regulatory factors to RNA Pol II required to regulate transcription and RNA processing (reviewed in^28,41^). Within this interaction the Mediator complex integrates general transcription factors and gene-specific trans-acting activators and repressors, presenting them to RNA Pol II to fine-tune responses^42–44^. The RNA Pol II CTD consists of conserved heptapeptide repeats which CTD-binding proteins recognize to regulate the transcription cycle and modulate RNA capping, splicing, and polyadenylation^28,41^. The phosphorylation and dephosphorylation of CTD Ser residues by various CTD kinases and phosphatases like CPL1 work to co-ordinate transcription and the recruitment of specific factors. The extent of CPL1’s interactions within the RNA Pol II-Mediator complex and its interaction with specific transcription factors and regulatory proteins to regulate the expression of target biotic and abiotic stress-responsive genes is not fully explored. It has been demonstrated CPL1 specifically dephosphorylates the Ser2 and Ser5 residues of RNA Pol II CTD^27,45^. Other relatively well studied Arabidopsis CPL proteins include CPL2, CPL3 and CPL4, where these proteins have CTD Ser5, Ser2, or Ser2 and Ser5 phosphatase activity respectively^27,46,47^. CPL2 is a regulator of plant growth and abiotic stress tolerance^48^, and a role for CPL4 as a negative regulator of xenobiotic detoxification has been demonstrated^46^. CPL3 also has a role in plant growth and interestingly analysis of *cpl3* mutants demonstrated CPL3 functions as a negative regulator of flowering and resistance against several leaf pathogens, the biotrophic powdery mildew fungus *Golovinomyces cichoracearum* and the bacterial pathogens *Pst*DC3000 and *P*. *syringae* pv. *maculicola*^35,47^. CPL1 is a positive regulator of flowering and we found no role for CPL1 in *PstDC3000* resistance suggesting the co-ordinated interaction of individual CTD phosphatases play unique roles in regulating biotic stress responses.

We found the *cpl1* mutants exhibited increased resistance to the root or leaf necrotrophic lifestyle fungal pathogens *F*. *oxysporum* and *A*. *brassicicola*, and increased tolerance to an insect pest. They also exhibited increased expression of JA-mediated *PR* defence genes. Intact JA-defences are required for resistance against *A*. *brassicicola* and the aphid pest *M*. *persicae*^14,37^ however, JA signalling plays contrasting roles in *F*. *oxysporum* disease symptom development. Up-regulation of JA-regulated defensive components leads to increased pathogen resistance, affirmed with our finding in the *cpl1* mutants, while global up-regulation of JA-signalling which includes senescence-related processes has an overriding effect and is linked to susceptibility. For example, mutations in downstream negative regulators such as the transcription factors MYC2 or ERF4 leads to increased expression of JA-defence genes and increased *F*. *oxysporum* resistance^49,50^. Conversely, mutations in upstream components such as the JA-coreceptor CORONATINE INSENSITIVE1 (COI1) or components of the Mediator complex (MED25/PFT1, MED18, MED20) abolish or reduce JA-sensitivity and signalling (JA-biosynthesis, defence, senescence) leading to resistance against *F*. *oxysporum* but increased susceptibility to leaf necrotrophs (*A*. *brassicicola*, *B*. *cinerea*)^10,14,16,51–53^. Mutation of another Mediator subunit, MED8, also increased resistance to *F*. *oxysporum* but it did not alter JA-signalling^10^. Combined with our new CPL1 findings, these examples highlight multiple levels of transcriptional and post-transcriptional control within the RNA Pol II-Mediator-co-regulator-transcription factor interaction to regulate responses to biotic stress. This is further exemplified by our finding that the CPL1 and ESR1/HOS5/RCF3 interacting proteins only co-regulate a small set of stress-responsive genes and that *esr1* mutants are not affected in JA-sensitivity^18^.

Using non-biased whole transcriptome RNA-seq we found genes up-regulated in *cpl1-8* were significantly enriched for processes relating to both biotic and abiotic stress. This included significant up-regulation of genes involved in heat shock, oxidative stress and redox control processes, and this was associated with enhanced tolerance to oxidative stress (Fig. 8). Genes involved in oxidative stress and reactive oxygen species (ROS)-signalling were also increased in the enhanced *F*. *oxysporum* resistant mutant *med20*^52^. Interestingly, the *F*. *oxysporum* resistant mutant *myc2* has reduced oxidative stress tolerance^54^. Opposing roles in redox processes and *F*. *oxysporum* disease resistance have also been proposed for genes encoding ROS producing NADPH oxidases *RESPIRATORY BURST OXIDASE HOMOLOGUES (RBOH*) and peroxidases (*PRX*) where *rbohd* and *prx33* mutants have increased disease resistance while a *rbohf* mutant has reduced resistance^55,56^. It has been suggested the plant oxidative burst in response to *F*. *oxysporum* infection may be advantageous to the pathogen^56^. In the case of *cpl1* mutants, the enhanced oxidative stress tolerance we observed may explain their increased tolerance to this pathogen. A microarray analysis of genes up-regulated in the *cpl1-2/fry2-1* mutant identified amongst others, two clusters of genes that overlapped with genes up-regulated in wild-type plants exposed to abiotic stresses or ABA^39^. This included several *LATE EMBRYOGENESIS ABUNDANT* (*LEA*) protein genes that we also identified in our *cpl1-8* up-regulated dataset. LEA proteins are associated with tolerance to abiotic stress where they are thought to act as cellular stabilizers and protectants of biomolecules and membranes^57^. Their increased expression in *cpl1* mutants likely contributes to their dual biotic and abiotic stress tolerance.

As with many class two pathogen susceptibility genes, *cpl1* mutants displayed an unwanted developmental characteristic (a delay in flowering) however, others have shown at least *F*. *oxysporum* disease resistance in roots and flowering time mediated in above ground tissues can be unlinked^52^. Apart from the delayed flowering phenotype, the *cpl1* mutants were otherwise phenotypically normal and do not show typical stressed phenotypes such as severely stunted growth or spontaneous lesions that exemplify many enhanced or constitutively activate defence or ROS signalling mutants^3–9^.

## Conclusions

We identify roles for CPL1 in biotic stress tolerance and provide additional insight into its regulatory network. We demonstrate CPL1 is involved in the negative regulation of oxidative stress that when lost leads to enhanced resistance to both fungal pathogens and insect pests. Our findings open a future line of research into the role of CPL1 in broader biotic stress responses and future work should aim to identify CPL1 interacting partners under biotic stress and their role in redox processes. In particular, how variations in *cpl1* alleles affect the degree of gain of function phenotypes and how this might be manipulated to tailor pathogen and pest resistance using useful alleles of plant susceptibility genes with a lack of unfavourable pleiotropic effects.

## Experimental Procedures

### Plant material and growth conditions

Unless otherwise specified, all experiments were conducted with the *Arabidopsis thaliana* Columbia-0 transgenic line JC66 which contains 791 bp of the *GSTF8* promoter fused to a luciferase reporter (*GSTF8:LUC*)^26^. Agar plate and soil grown plants were incubated under a long day 16-h light/8-h dark cycle at 22 °C. Seeds were surface-sterilized, stratified at 4 °C, then plated onto 100-mm square agar plates containing Murashige and Skoog (MS) salts or onto soil as described previously^25^. Mutagenesis of wild-type *GSTF8:LUC* and identification of constitutive *GSTF8:LUC* mutants was described previously^18,23^. The EMS mutant *cpl1-2/fry2-1* (CS24934) and T-DNA insertion mutants *cpl1-1* (CS6541) and *myc2* (SALK_061267C) were obtained from the Arabidopsis Biological Resource Centre (ABRC). For generation of plants expressing the wild-type *CPL1* gene *At4g21670*, 1010 bp of the *CPL1* promoter and coding sequence were amplified off genomic DNA using primers listed in Supplemental Table S4. The resulting amplicon was cloned into pDONORZeo, moved into the binary vector pB7WG and confirmed by sequencing. The *CPL1p:CPL1_*B7WG construct was mobilized into *Agrobacterium tumefaciens* GV3101 and transformed into wild-type, *esr3-1/cpl1-7* and *esr3-2/cpl1-8* using standard floral dip techniques. Transgenic T_1_ plants were selected based on resistance to 10 µg/mL glufosinate ammonium (Fluka).

### Bioluminescence and luciferase assays

Plates for luciferase assays were supplemented with 50 µM luciferin (Biosynth AG). Seedling bioluminescence was captured and quantified as previously described^18,58^ using a Nightowl or Nightshade molecular light imager (Berthold Technologies) with Winlight32 (v 2.7) or IndiGo (v 2.0.3.0) software (Berthold Technologies) respectively.

### Mapping, DNA isolation, Illumina sequencing, assembly and SNP annotation

For allelism tests, reciprocal crosses between mutants or wild-type plants were conducted and bioluminescence activity in F_1_ progeny assessed. For initial mapping a genetic cross between the *esr3-1/cpl1-7* or *esr3-2/cpl1-8* mutants and *Ler* were generated and mapping conducted on 40 or 104 homozygous *cpl1-7* or *cpl1-8* F_2_ plants respectively (exhibiting constitutive *GSTF8:LUC* activity) with a set of 18 simple sequence-length polymorphism (SSLP) markers as described previously^18^. The 18 SSLP markers are evenly spaced over the 5 Arabidopsis chromosomes with each chromosome represented by 3–4 markers spaced approximately at 20–25 cM intervals. For whole genome sequencing of individual wild-type *GSTF8:LUC*, *cpl1-7* or *cpl1-8* plants, Illumina Truseq DNA libraries were generated using manufactures recommendations on CTAB extracted DNA, sequenced on an Illumina HiSeq. 1000 platform, and reads cleaned, trimmed, mapped against the TAIR10 release of the Arabidopsis genome, and SNPs called as described previously^18^. For Next Generation mapping a three times backcrossed *cpl1-7* line was crossed with *Ler*, pooled DNA (CTAB extraction) from 53 homozygous *cpl1-7* F2 plants were sequenced at 60–70x coverage by the Australian Genome Research Facility (AGRF) using an Illumina HiSeq Platform. 82.7 million paired-end reads (100 bp in length) were cleaned, trimmed and mapped to the Arabidopsis TAIR10 genome reference sequence, SNPs called using the recommended SAMtools mpileup script and processed through the NGM tool http://bar.utoronto.ca/ngm/^59^ as described previously^18^.

### Pathogen and pest assays

The isolates and inoculations using *F*. *oxysporum* (*Fo*5176), *R*. *solani* (AG8), *Pst* (DC3000) and *A*. *brassicicola* (UQ4273) were performed as described previously^16,23,60^. Infestation assays with *M*. *persicae* were performed as previously described^61^. Briefly, four week old plants of similar size were individually caged in plastic bottles and infested with 20 aphids per plant. Mock treated plants were caged in bottles only. At 14 days post infestation, each leaf was assessed for aphid-induced symptoms and given a score from 0–4 (0 = no symptoms; 1 = <¼ chlorotic; 2 = ½ chlorotic; 3 = >½ chlorotic or necrotic, 4 = whole leaf necrotic).

### MeJA root elongation, methyl viologen and salt inhibition assays

For MeJA root elongation inhibition assays seeds were sterilized and plated onto MS media in either the presence or absence of 25 or 50 µM MeJA (Sigma). Root length was measured on 7-day old seedlings using ImageJ^62^. For Methyl viologen (MV) assays, seeds were sterilized and plated onto MS media in either the presence or absence of 0.5 or 1 µM MV (Sigma-Aldrich) and the number of germinated seedlings with green, fully emerged cotyledons determined at 10-days. For salt assays, seeds were sterilized and plated onto petri dishes with filter paper laden with 50 mM NaCl or sterile water. Seedlings were scored for germination and emergence of green cotyledons at 14 days.

### RNA isolation, RNAseq and qRT-PCR

For RNA-seq and follow-up qRT-PCR experiments on untreated plants, tissue was collected from whole 12-day old seedlings germinated and grown upright on MS plates. Three separate biological replicates were taken for each genotype with each replicate consisting of tissue pooled from 15–20 seedlings grown at the same time in the same environment, then frozen in liquid nitrogen and stored at −80 °C. RNA isolation was performed using the Qiagen RNeasy Plant Mini Kit (Qiagen) followed by DNase treatment using TURBO DNase (Ambion). For RNA sequencing, Illumina TruSeq libraries were generated from mRNA derived from 1 µg of total RNA, and 100 bp paired ends sequenced on a HiSeq1000 platform (Illumina) over 4/10 of two lanes. RNA-seq data was processed and analysed as described previously^18^. Briefly, RNA-seq paired-end reads were sorted into pairs and singleton’s after trimming for low-quality base-calls, Illumina adapter sequences and removal of short reads. Around 60 million paired end reads for each library were mapped to the TAIR10 Arabidopsis genome reference via Tophat (v2.0.9)^63^, normalised, and significantly differentially expressed transcripts between wild-type and *cpl1-8* calculated using Cuffdiff (Cufflinks v2.1.1^64^) with default Benjamini-Hochberg correction for multiple-testing (based on a False Discovery Rate ≤0.05). Functional annotations of genes and AGI symbols were sourced from TAIR10 datasets. For discovery of novel genes and isoforms Cufflinks (v2.2.2) was run as above but with an overhang-tolerance of 20 bp and the guided reference annotation setting GTF-guide. Cuffdiff (Cufflinks v2.2.2) was used to identify alternate splicing and promoter usage using a merged transcriptome (Cuffmerge) from each new assembly. RNA-seq reads have been deposited in the NCBI Sequence Read Archive under BioProject ID PRJNA421838. For qRT-PCR, complementary DNA synthesis and analysis was performed as described before using validated β-actin reference genes^18^. Gene expression was calculated using the equation: relative ratio gene of interest/actin = (Egene^−Ct gene^)/(Eactin^−Ct actin^) where Ct is the cycle threshold value. Gene-specific primer sequences are listed in Supplemental Table S4.

### Classification of DEG RNA-seq data

Gene Ontology (GO) term enrichment analysis of RNA-seq DEGs was performed using agriGO v1.2^65,66^ with the default FDR (p < 0.05). DEGs were mapped onto Arabidopsis biotic stress pathways using MapMan v3.5.1^38^. Identification of overlapping DEGs between *cpl1-8* and *esr1-1* was performed using jvenn^67^.

## Electronic supplementary material

Supplementary Information

Supplemental Tables

## Data Availability

RNA-seq reads associated with this manuscript are available in the NCBI Sequence Read Archive under BioProject ID PRJNA421838.
