# Supplementary material for: The Arabidopsis RNA Polymerase II Carboxyl Terminal Domain (CTD) Phosphatase-Like1 (CPL1) is a biotic stress susceptibility gene
Source: Sci Rep. 2018 Sep 7;8:13454. doi: 10.1038/s41598-018-31837-0 (PMC6128934; doi:10.1038/s41598-018-31837-0)
Supplement: Supplementary file 1 — Supplementary Information [file 41598_2018_31837_MOESM1_ESM.pdf]

SUPPLEMENTARY INFORMATION

The Arabidopsis RNA Polymerase II Carboxyl Terminal Domain (CTD) Phosphatase-Like1 (CPL1) is a biotic stress susceptibility gene

Louise F. Thatcher[1*], Rhonda Foley[1], Hayley J. Casarotto[1], Ling-Ling Gao[1], Lars G. Kamphuis[1,2], Su Melser[1#], Karam B. Singh[1,2]

[1] CSIRO Agriculture and Food, Centre for Environment and Life Sciences, Floreat, Western Australia, Australia

[2] Centre for Crop and Disease Management, Department of Environment and Agriculture, Curtin University, Bentley, Western Australia 6102, Australia

Present address: #Su Melser, INSERM U1215, Université de Bordeaux, NeuroCentre Magendie, Bordeaux, France.

Corresponding author email: * Louise.Thatcher@csiro.au

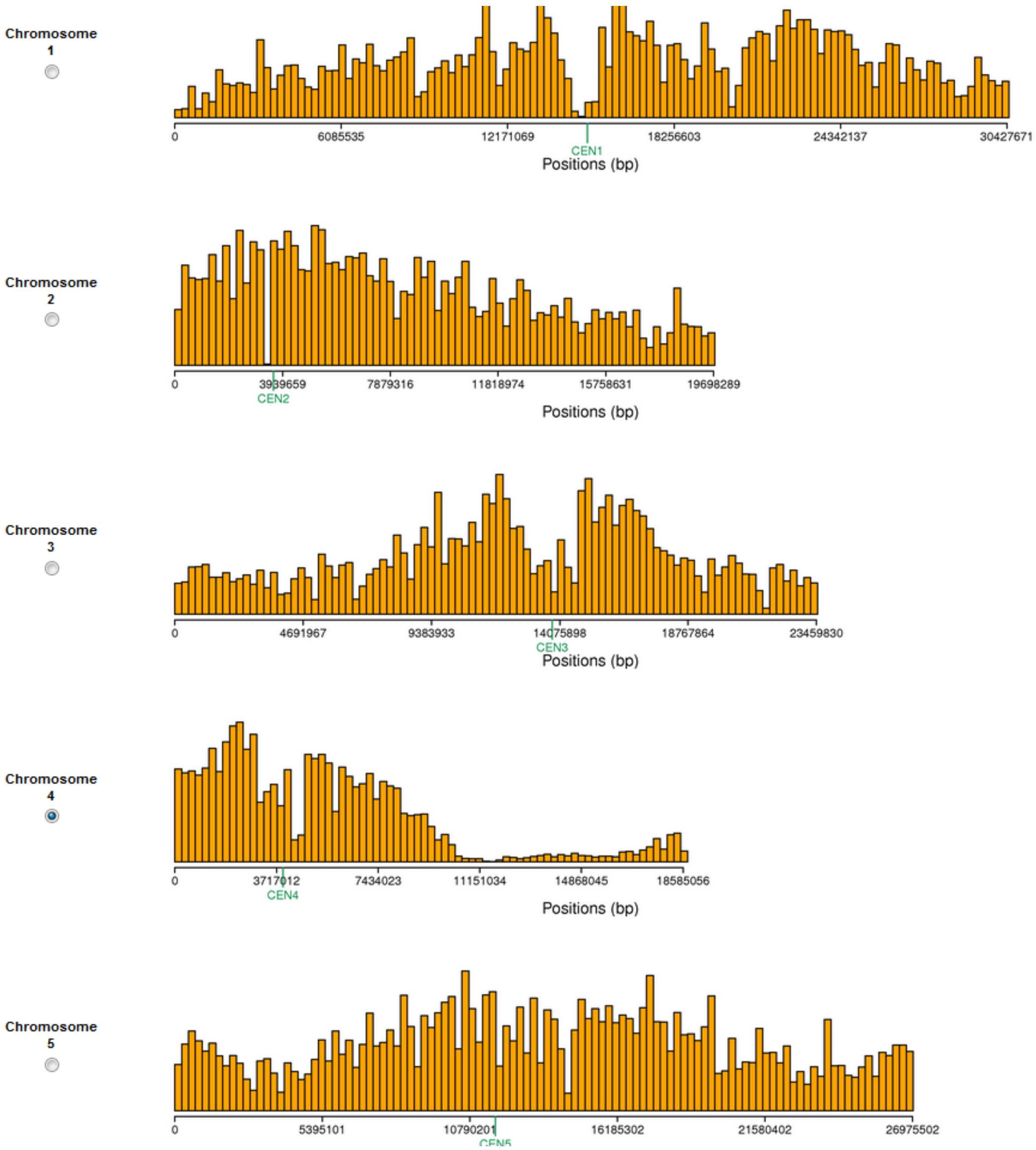

Supplemental Figure S1. Next Generation Mapping locates *esr3-1* locus to a region on chromosome 4. Whole-genome sequencing of homozygous *esr3-1* F_2s from an *esr3-1* and Ler outcross coupled with the Next Generation Mapping tool identified SNP desserts (depression on chromosome 4) corresponding to *esr3-1* linkage. Shown are genome-wide SNP frequencies (y-axis) plotted as a function of chromosomal position (x-axis) using a bin size of 250 kb.

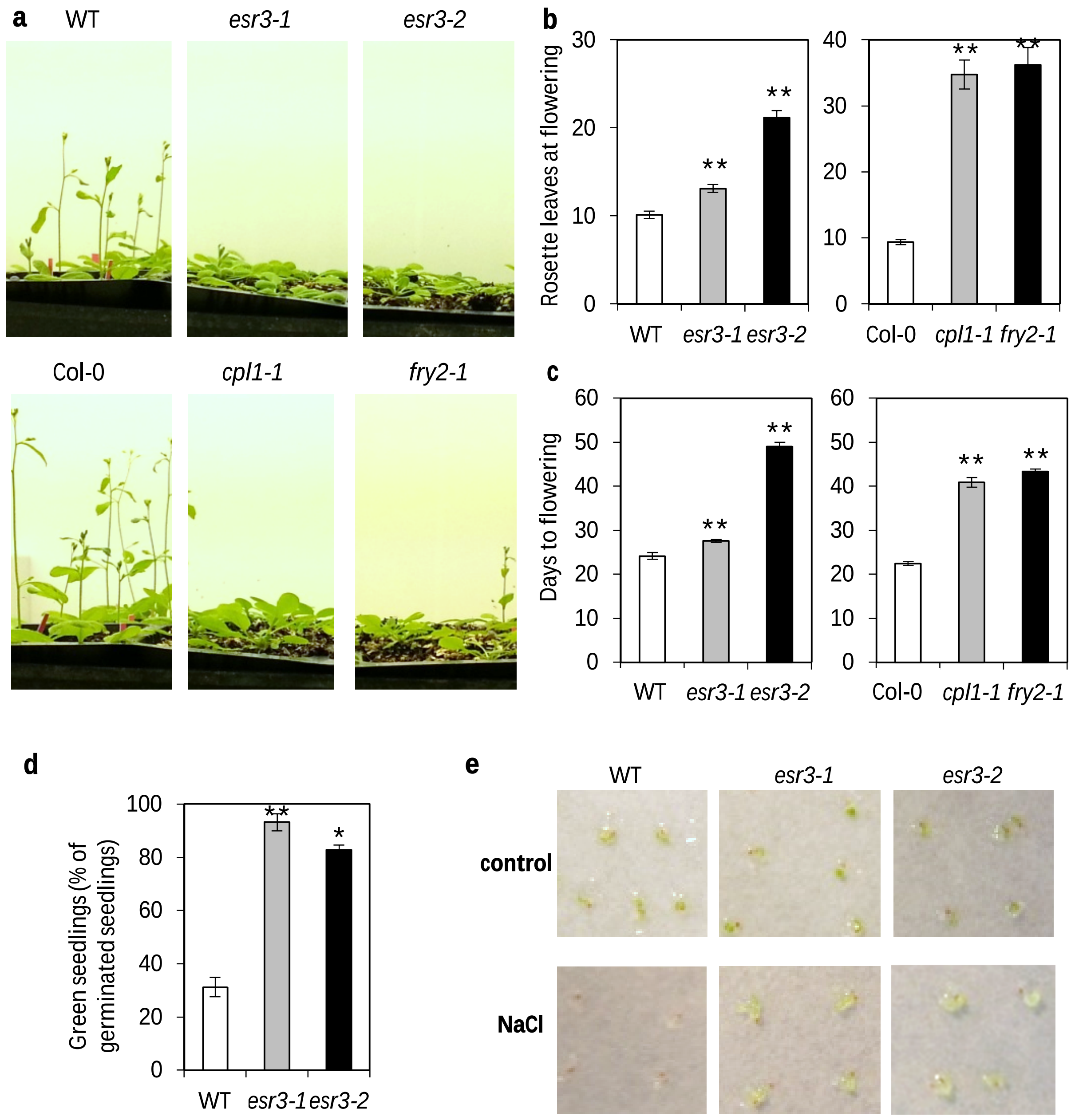

Supplemental Figure S2. The *esr3* alleles complement *cpl1* phenotypes. (a-c) Side-by-side comparisons of *cpl1* mutant development confirms delayed flowering in *esr3*, *cpl1-1* and *fry2-1/cpl1-2* mutants. Shown are (a) representative plants at 28 days of age, (b) number of rosette leaves at flowering and (c) number of days to flowering. Wild-type (WT) *GSTF8::luc* and Col-0 are controls for *esr3* and *cpl1* mutants respectively. Plants were grown under long day conditions. Values are averages ± SE (n=8). (d-e) *esr3* mutants have increased tolerance to NaCl (50mM). Seedlings were grown on NaCl or water (control) laden filter paper for 14 days. (d) Percentage of germinated seedlings with green cotyledons as depicted in (e) representative images. Values are averages ± SE (n=51-62). Asterisks indicate values that are significantly different (***P* <0.01, **P* <0.05 Student's *t*-test) from control treatment. Similar results were obtained in an independent experiment.

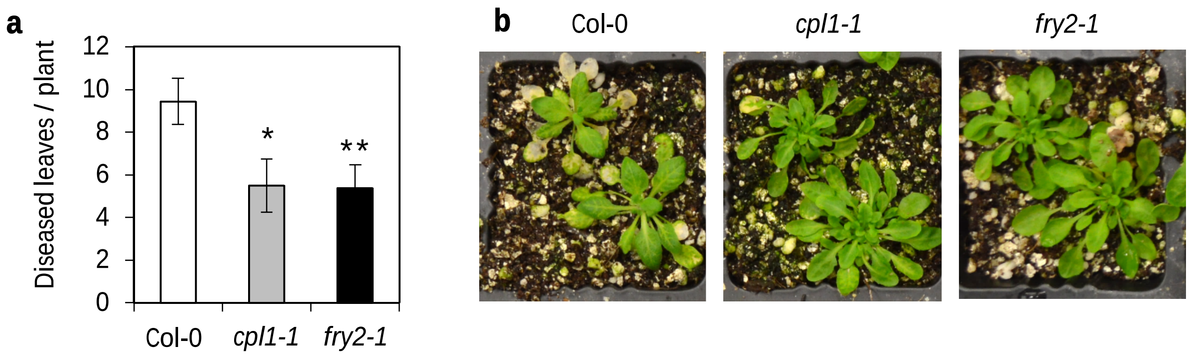

Supplemental Figure S3. The *cpl1-1* and *cpl1-2/fry2-1* alleles complement *the cpl1-7* and *cpl1-8 Fusarium* wilt disease phenotypes. (a-b) Disease phenotypes of *F. oxysporum* inoculated plants with (a) diseased leaves and (b) representative images of plants 21 days post inoculation. Values are averages ± SE (n=16). Asterisks indicate values that are significantly different (***P* <0.01, **P* <0.05 Student's *t*-test) from controls. Similar results were obtained in independent experiments.

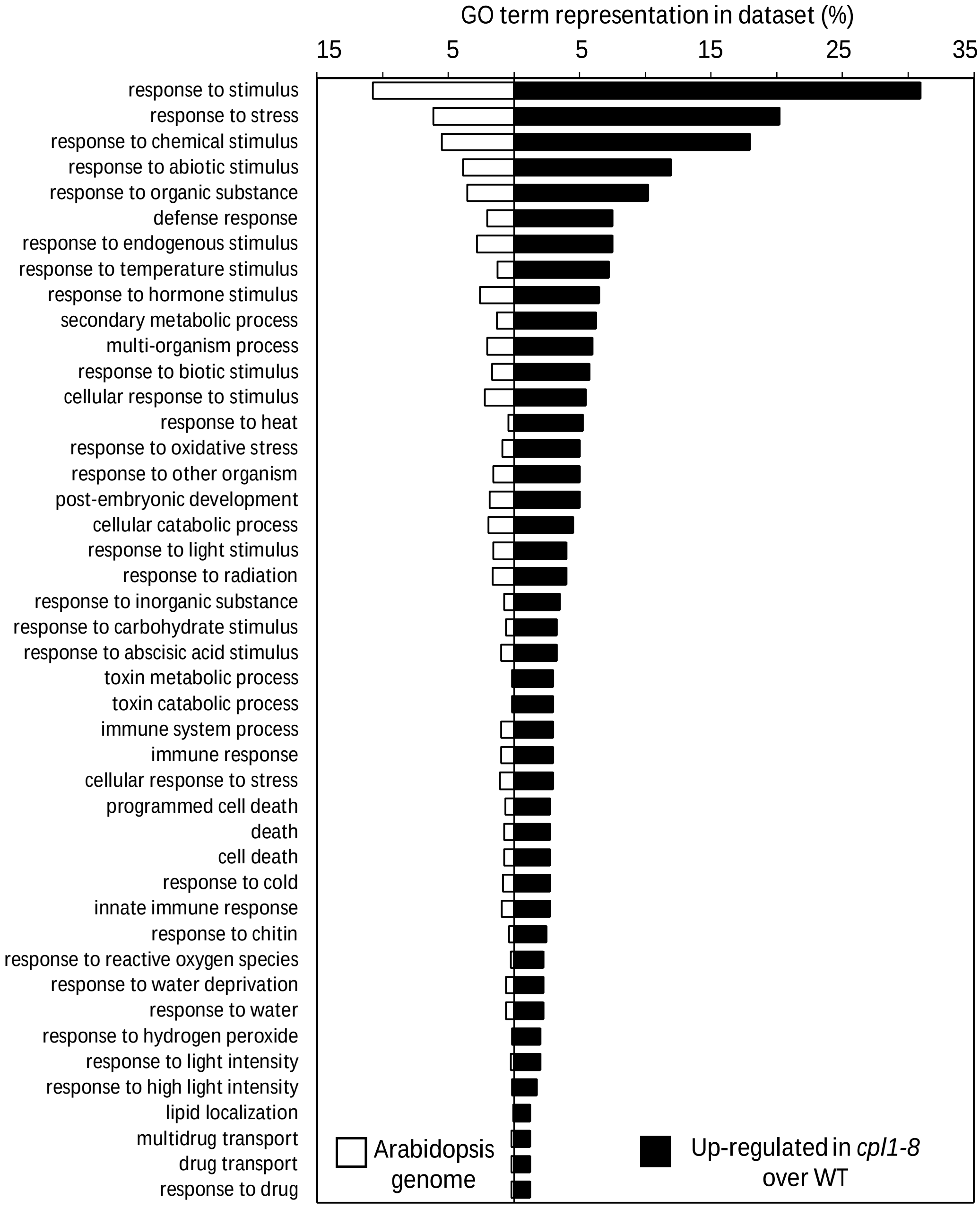

Supplemental Figure S4. Significant enrichment of stress, redox and defense-related biological process Gene Ontology (GO) terms in *cpl1-8* up-regulated genes. ≥2-fold significantly up-regulated genes in *cpl1-8* (compared to wild-type) were analysed for enrichment of GO terms associated with biological processes. Shown are GO term representations in the *cpl1-8*/WT dataset compared to representation in the whole Arabidopsis genome with a *P* value <0.05 adjusted by the False Discovery Rate.

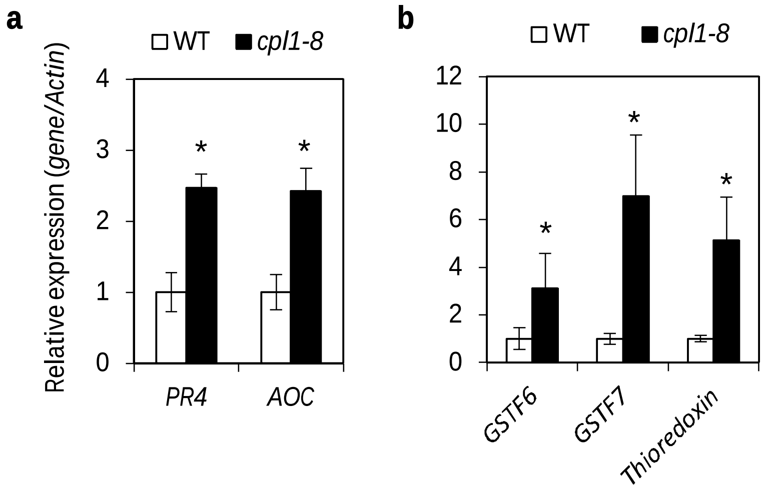

Supplemental Figure S5. Confirmation of representative *cpl1-8* up-regulated genes by qRT-PCR.

Expression confirmation of subset of up-regulated genes in *cpl1-8* compared to wild-type (WT). (a) JA-regulated defence and JA-biosynthesis marker genes. (b) Redox-associated marker genes. Shown are values from 12 day old seedlings (values are averages ± SE of 3 biological replicates consisting of pools of 15-20 seedlings). Gene expression levels are relative to the internal control β-actin genes. Asterisks indicate values that are significantly different (*P <0.05 Student's t-test) from WT.

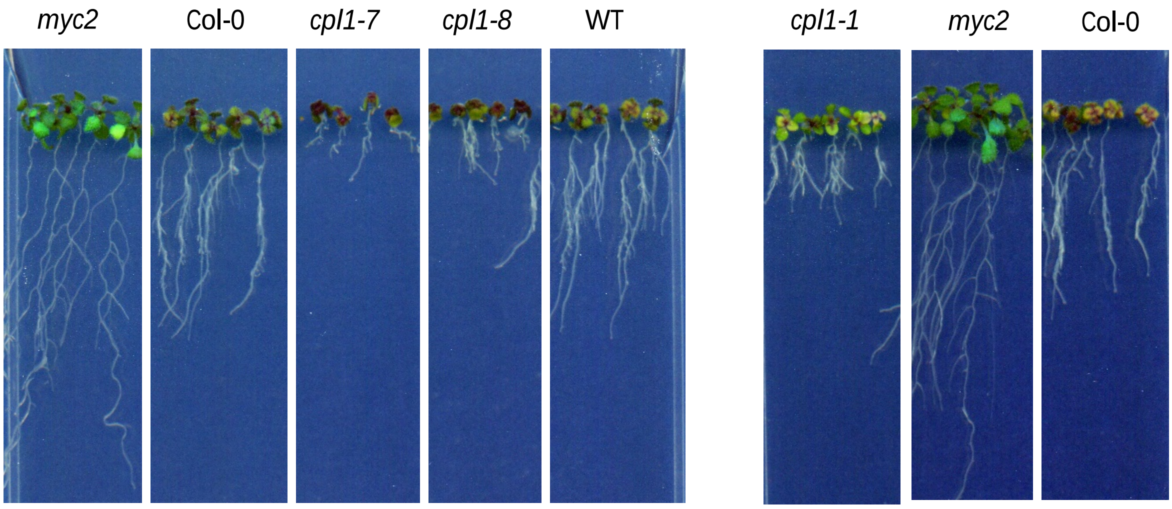

Supplemental Figure S6. Sensitivity of *cpl1* alleles to MeJA. Sensitivity of wild-type (WT, *GSTF8::luc*), *cpl1-1, cpl1-7* and *cpl1-8* seedlings to JA was determined by MeJA inhibition of root growth on media containing MeJA (50 uM). Col-0 and the JA-insensitive mutant *myc2* included as controls.

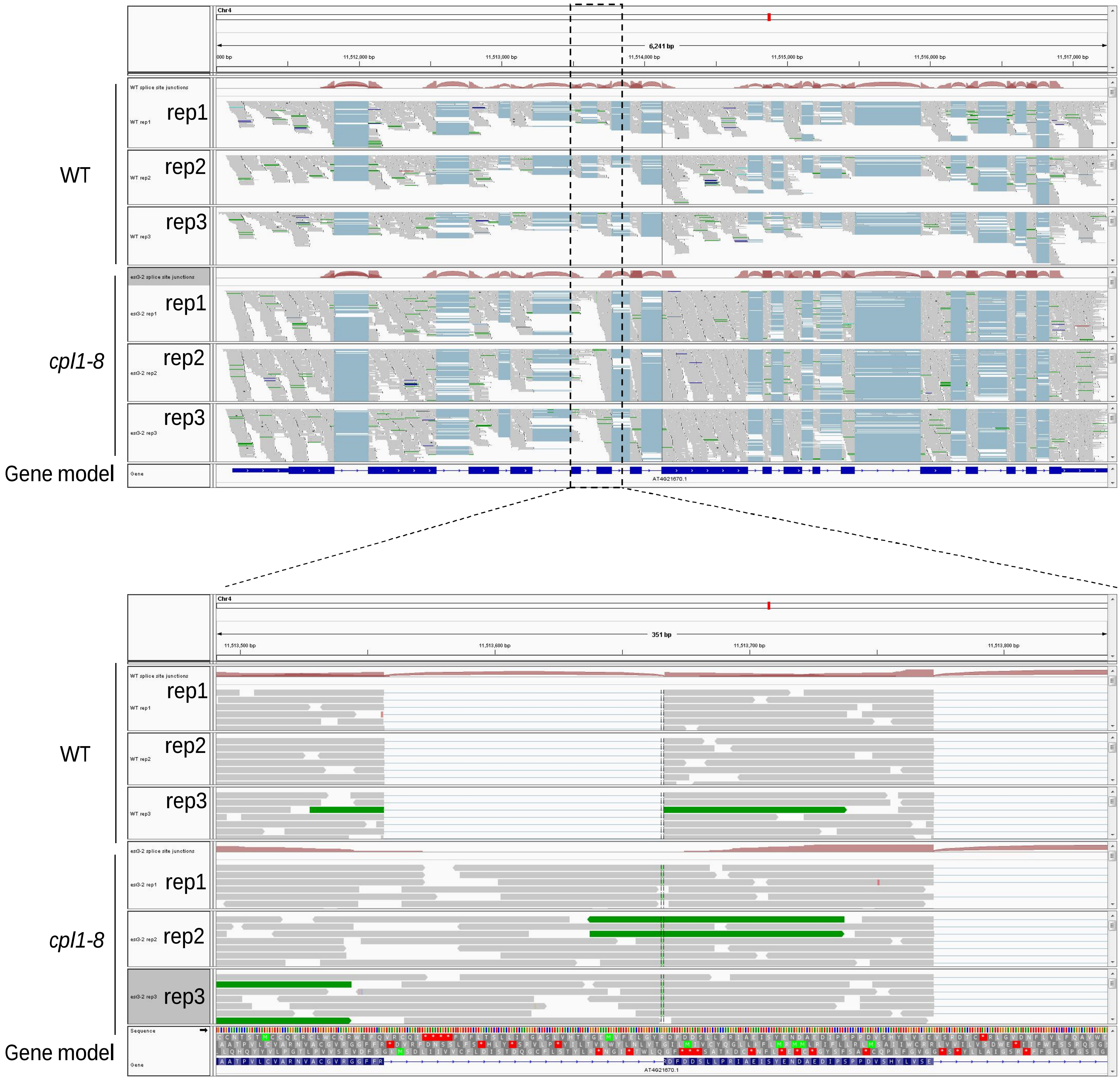

Supplemental Figure S7. Misplicing in *cpl1-8*. Mapping of RNAseq reads from three biological replicates for each genotype identifies misplicing in *cpl1-8* at the 5' boundary of the 6th exon. The bottom image is an enlarged region of the mispliced intron.
